# Enhanced force production in old age is not a far stretch: an investigation of residual force enhancement and muscle architecture

**DOI:** 10.1002/phy2.4

**Published:** 2013-06-07

**Authors:** Geoffrey A Power, Demetri P Makrakos, Charles L Rice, Anthony A Vandervoort

**Affiliations:** 1 Human Performance Laboratory, University of CalgaryCalgary, Alberta, Canada; 2 Canadian Centre for Activity and Aging, School of Kinesiology, Faculty of Health Sciences, The University of Western OntarioCanada; 3 Department of Anatomy and Cell Biology, The University of Western OntarioCanada; 4 School of Physical Therapy, Faculty of Health Sciences, The University of Western OntarioCanada

**Keywords:** Aging, eccentric, EMG, fascicle

## Abstract

In older adults, isometric force production is enhanced following a voluntary lengthening contraction when compared with isometric force produced at the same muscle length without a prior lengthening contraction. This phenomenon is termed residual force enhancement (RFE), and appears to be related to the age-related maintenance of eccentric (ECC) strength. However, it is unknown whether age-related changes in muscle architecture contribute to greater RFE at short and long muscle lengths in old age. Neuromuscular properties of the knee extensors were assessed on a HUMAC NORM dynamometer. Torque was examined in young (26 ± 3 year, *n* = 11) and old men (77 ± 6 year, *n* = 11) during brief maximal voluntary isometric contractions (MVC) at 80° and 120° (180° representing full knee extension) and then compared with torque during a steady-state phase at the same joint angle following a maximal voluntary lengthening contraction at 30°/sec over a 60° joint excursion; either from 140 to 80° (long), or from 180 to 120° (short). Ultrasound images were obtained from the vastus lateralis during the isometric phase for each condition. When comparing the ECC torque with the MVC isometric torque, old men had 17% greater ECC:MVC ratios than young men, confirming an age-related maintenance of ECC strength. The extent of RFE was greater at long versus short but independent of age. At rest, old had shorter (∼18%) and less pennated (∼22%) fascicles. However, changes in fascicle length and pennation during contraction did not contribute to RFE in either group. Thus, age-related changes in muscle architecture may not contribute to RFE.

## Introduction

The loss of isometric strength is a well-documented consequence of natural adult aging (Vandervoort 2002; Narici and Maffulli 2010). Conversely, strength during lengthening muscle actions (eccentric; ECC) is well maintained into old age (Vandervoort et al. 1990; Roig et al. 2010; Power et al., 2012c). We found in older adults that isometric force production following a lengthening contraction was greater than isometric force produced at the same muscle length prior to the stretch (Power et al., 2012b). This phenomenon, observed in humans and in reduced preparations, is termed residual force enhancement (RFE) (see Campbell and Campbell 2011 for review). In older adults, RFE is in part, related to the maintenance of ECC strength (Power et al., 2012b). However, this maintenance alone cannot explain entirely the greater RFE in old compared with young adults. Thus, we aimed to investigate whether age-related changes in muscle architecture contribute to RFE at short and long muscle lengths in old age.

Muscle force production is dependent highly upon prior activation (McGowan et al. 2013) in that, isometric force of an activated skeletal muscle during a steady-state isometric phase following stretch is greater than isometric force produced at the same muscle length prior to stretch (Abbott and Aubert 1952; Rassier et al. 2003). This residual force enhancement can last for up to 30 sec following stretch (Abbott and Aubert 1952), although for most investigations of human electrically stimulated (de Ruiter et al. 2000; Lee and Herzog 2002) and voluntary contractions, RFE dissipates within 10 sec (Lee and Herzog 2002; Pinniger and Cresswell 2007; Oskouei and Herzog 2009; Power et al., 2012c), and is particularly short lived (Hahn et al. 2010; Seiberl et al. 2010; Shim and Garner 2012) or absent (Hahn et al. 2007) when investigated in larger muscle groups such as the knee extensors; warranting further investigation of this particular model. A combination of both active and passive structural properties of muscle force generating and transmitting structures (Herzog and Leonard 2002) contribute to RFE. Active force enhancement is observed along the entire force-length (F-L) relationship and is thought to occur through an increase in the average force produced by each cross-bridge (Lee and Herzog 2002), combined with an increased proportion of strongly bound cross-bridges (Rassier et al. 2003). Passive force enhancement (PFE) is related primarily to length-dependent properties of muscle, occurs on the descending limb of the F-L relationship and is associated with the engagement of passive viscoelastic elements and sarcomere length nonuniformities during and following stretch (Edman et al. 1982; Herzog and Leonard 2002; Rassier 2012). Most notably, RFE is greater when stretch is applied on the descending limb (long muscle length) versus the plateau or ascending limb (short muscle length) of the F-L relationship owing to the greater contribution of passive elements (Edman 2012), although this is currently unknown in old age.

Impaired isometric force production in older adults has been attributed to a reduction in the number of viable cross-bridges and average force produced per bridge (D'Antona et al. 2003). On the other hand, older adults benefit greatly during (Ochala et al. 2006) and following stretch to enhance subsequent torque production (Power et al., 2012b). In our previous study (Power et al., 2012b), succeeding a conditioning stretch, both old and young adults followed the typical exponential decline in force (Edman 2012), but for older adults the time to reach an isometric steady-state force level was longer when compared with young. The longer time to steady state and elevated PFE in older than younger adults indicated that passive structural mechanisms, potentially affecting musculotendinous stiffness, may have a disproportionately greater contribution to force enhancement in old age (Power et al., 2012b). Depending on where along the F-L relationship stretch is applied, force production and RFE in older adults could be enhanced more than in young.

In young adults, stretch-induced changes in fascicle length and pennation angle of the knee extensors and ankle dorsiflexors did not contribute to RFE (Seiberl et al. 2010; Tilp et al. 2011). With adult aging, however, muscle fascicles become shorter and less pennated, and these architectural changes can contribute up to 40% of age-related force loss (Narici et al. 2003; Narici and Maffulli 2010). Hence, muscles of older adults with shorter fascicles may fall further along the descending limb of the F-L relationship for a given amount of stretch than those of young adults, optimizing the contribution of passive force transmitting elements. Therefore, older adults could benefit from greater stretch amplitude effectively increasing RFE on the descending limb (long muscle length) of the F-L relationship, owing mainly to the greater viscoelastic components contributing to RFE.

Accordingly, the purpose of this study was to investigate RFE and changes in muscle architecture of the knee extensors in young and old men at short and long muscle lengths, which fall on the ascending and descending limbs of the F-L relationship (Hahn et al. 2007; Shim and Garner 2012), respectfully. It is hypothesized that due to the age-related increase in RFE, older adults will exhibit greater RFE compared with young, particularly over the descending limb of the F-L relationship (i.e., long muscle length) owing to a greater contribution of PFE. Finally, due to age-related changes in muscle architecture impairing force production, when tested during the steady-state phase following the conditioning stretch, older adults may benefit from a stretch-induced increase in fascicle length to enhance force production compared to the reference isometric condition.

## Material and Methods

### Participants

All young (*n* = 11, 25.7 ± 2.6 year, 176.8 ± 4.7 cm, 79.2 ± 10.9 kg) and old men (*n* = 11, 76.8 ± 5.7 year, 173.9 ± 5.9 cm, 80.6 ± 9.3 kg) were asked to refrain from unaccustomed and strenuous exercise 2 days prior to testing and not to consume caffeine within 4 h prior to testing. All participants were recreationally active with no known neurological or musculoskeletal conditions. The young adults were recruited from the university population and the older adults were recruited from a local senior's fitness group consisting of walking, and light calisthenics three times per week. This study was approved by the local University's Review Board for Research Involving Human Subjects and conformed to the Declaration of Helsinki. Informed written consent was obtained from all participants prior to testing.

### Experimental arrangement

All testing was conducted on a HUMAC NORM dynamometer (CSMi Medical Solutions, Stoughton, MA). The dominant leg was secured tightly to the dynamometer leg attachment with inelastic straps, aligning the lateral epicondyle of the knee with the rotational axis of the dynamometer. Extraneous movements were minimized using nonelastic shoulder, waist, and thigh straps. Participants sat in a slightly reclined position with the hip angle set at ∼100°. All baseline voluntary and evoked isometric contractions were performed at a knee joint angle of 100° knee extension (KE) (referenced to a straight knee of 180°). For the determination of RFE, isokinetic (30°/sec) lengthening contractions began at 140° KE until 80° for the *LONG (descending limb)* muscle length condition and 180° KE until 120° for the *SHORT (ascending limb)* muscle length condition. Therefore, stretch was applied through a 60° joint excursion in both conditions.

### Electromyography

Electromyography (EMG) was collected using self-adhering Ag-AgCl surface electrodes (1.5 × 1 cm; Kendall, Mansfield, MA). Prior to electrode placement, the skin was cleaned aggressively with presoaked alcohol swabs. A bipolar electrode configuration was used with the active and reference electrodes positioned 1 cm apart over the muscle belly (midpoint of the thigh from the greater trochanter to the lateral epicondyle) of the vastus lateralis (VL) and a ground electrode was placed over the patella.

### Experimental procedures

Isometric stimulated contractions of the quadriceps femoris muscle group were evoked electrically through two custom-made aluminum electrode pads secured transversely over the anterior thigh with cloth tape. These electrodes were wrapped in a conductive gel-soaked paper towel layer and one electrode was positioned ∼7 cm distal to the greater trochanter of the femur and the other was positioned ∼9 cm distal to the inferior edge of the proximal stimulating electrode with the knee joint angle of 100°. Depending on the size of the thigh and to ensure the greatest muscle mass activation of knee extensors without activation of antagonist muscles, the stimulating electrode pad sizes varied between 8–11 cm in width and 16–20 cm in length. Visual inspection and palpation were used to ensure that only the knee extensors were activated by the electrical stimulation. A computer-triggered stimulator (model DS7AH, Digitimer, Welwyn Garden City, Hertfordshire, UK) set at 400 V provided the electrical stimulation using a pulse width of 200 μs. Knee extensor peak twitch torque (*P*_t_) was determined by increasing the current until a plateau in *P*_t_ was achieved, and then the current was further increased by at least 15% to ensure maximal activation.

**Figure 1:**
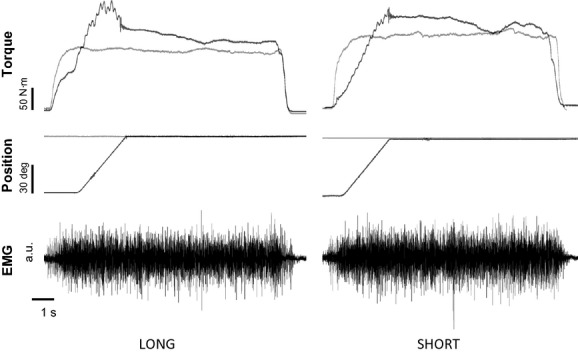
Unprocessed exemplar data depicting the determination of residual force enhancement (RFE) at LONG and SHORT muscle lengths in an older subject. Passive force enhancement (PFE) was determined to be an elevated force above resting baseline succeeding relaxation after stretch.

A 3–5 sec isometric baseline maximal voluntary contraction (MVC) was performed. To ensure MVC attempts were maximal, participants were provided visual feedback of the torque tracing on a computer monitor, and were exhorted verbally during all voluntary efforts. Voluntary activation was assessed using the modified interpolated twitch technique (Gandevia 2001). The amplitude of the interpolated torque evoked during the peak plateau of the MVC was compared with a resting *P*_t_ evoked 1 sec following the MVC when the muscles were relaxed fully. Voluntary activation was calculated as percent voluntary activation (%) = [1 − interpolated *P*_t_/resting *P*_t_] × 100%. Values from the MVC with the highest peak torque were recorded. The protocol used to determine residual force enhancement (Fig. 1) involved a 10 sec isometric reference MVC at either 80° (*LONG*) or 120° (*SHORT*) of KE followed by 3 min of rest and then maximal activation of the knee extensors for a total of 10 sec, consisting of 1 sec at either 140° (*LONG*) or 180° (*SHORT*) KE, followed by a 2 sec stretch at 30°/sec, and ending with a 7 sec isometric MVC at the same knee angle as the reference MVC (either 80° or 120°) (Fig. 1). Eccentric strength (ECC) was determined as the torque amplitude during stretch recorded at 100° KE. Finally, an isovelocity maximal voluntary shortening contraction (15°/sec) was performed through the entire range of motion to determine concentric (CON) strength recorded at 100°, and the optimal angle of torque production.

### Ultrasound image collection

To investigate the effects of age-related changes in muscle architecture on RFE ultrasound imaging was performed on a subset of 10 representative participants [five young (27 ± 3 year, 177.0 ± 2.9 cm, 86.9 ± 3.3 kg) and five old (77 ± 6 year, 175.6 ± 7.4 cm, 79.4 ± 8.0 kg) men]. Ultrasound images were collected via a linear array probe (GE model M12L, 4.9 mm, 5–13 MHz), using a Vivid 7 system (GE Healthcare, Mississauga, Ontario, Canada). Images were collected at rest, during the isometric reference MVC, and during the isometric steady-state MVC following stretch at either the *SHORT* or *LONG* muscle length conditions. The probe was placed over the VL muscle at approximately 50% of the length of the femur between the lateral epicondyle and the greater trochanter. Once a suitable placement, which yielded the best image was determined, the location was marked on the subject's skin using indelible ink. The probe was held secured firmly in place by the same operator for all tests.

### Data reduction and analysis

**Figure 2:**
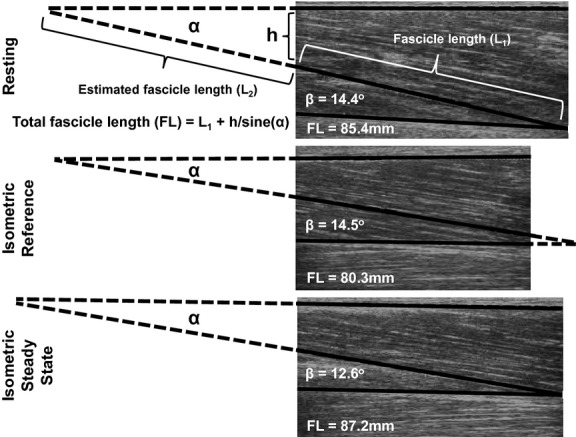
Ultrasound images from a representative older adult at LONG muscle length showing fascicle length (FL) and angle of pennation (β) measurement at rest, during the isometric reference MVC, and during the isometric steady state following lengthening. The solid lines represent the superficial and deep aponeuroses. Pennation angle (β) is the angle at which a fascicle leaves the deep aponeurosis and intersects the theoretical superficial aponeurosis indicated via the extended broken line. Fascicle length was calculated as the sum of the measured fascicle length (L_1_) and the estimated (L_2_) fascicle length [h/Sine(α)].

Torque and position data were sampled by the dynamometer at a rate of 500 Hz. All data were converted to digital format using a 12-bit analog-to-digital converter (model 1401 Power, Cambridge Electronic Design, Cambridge, UK). Residual force enhancement was calculated by determining the mean torque value over 1 sec epochs during the reference MVCs, divided into the mean torque value for 1 sec during the steady state of the MVC following the end of stretch corresponding to the same time point and muscle length as the reference MVC (Fig. 1). Residual force enhancement was analyzed over the final 6 sec of the isometric reference and corresponding isometric steady-state phase and averaged over each 1 sec time epoch. Isometric steady state was defined as when the torque value following stretch reached a statistically significant difference from the first time epoch and force transients were no longer present. Residual force enhancement was defined as the percent difference in isometric torque following stretch relative to the reference MVC at the same muscle length. The EMG signals were preamplified (×100), amplified (×2), band-pass filtered (10–1000 Hz), and sampled online at 2500 Hz using Spike 2 software (version 7.07, Cambridge Electronic Design Ltd). The EMG for the VL muscle was recorded during the reference MVC and was expressed as a root mean square (RMS) value over each 1 sec epoch. Following stretch, EMG was analyzed over each of the six 1 sec epochs. All subsequent RMS values of EMG during voluntary contractions were normalized to the EMG RMS value corresponding to 1 sec about peak MVC torque amplitude obtained during baseline. Torque-length relationships (T-L) were constructed from a 15°/sec isovelocity shortening contraction. The torque values were averaged over intervals of 5° and normalized to the peak torque amplitude achieved during the contraction. These data were then fitted with a third order polynomial (*R*^2^ = 0.99 for both young and old). Spike 2 software (Version 7.07) was used off line to determine torque values of all contractions. All ultrasound images captured during testing were transferred to a desktop computer for offline analysis using EchoPAC software (v.7.0.1, GE Vingmed Ultrasound) which allowed for the calculation of pennation angles and fascicle lengths. Pennation angle was defined as the angle between the fascicle and the deep aponeurosis. Fascicle length was defined as the length of a line coincident with the fascicle, between the deep and superficial aponeuroses. Images were selected so that fascicles were visible near the deep aponeurosis, however, the fascicle was often not visible in its entirety in which case its intercept with the superficial aponeurosis had to be extrapolated (Reeves and Narici 2003) as illustrated in Figure 2.

### Statistical analysis

Using SPSS software (version 16, SPSS Inc. Chicago, IL) unpaired t-tests were performed to compare baseline differences in neuromuscular function between young and old. A two-way analysis of variance (Age × Muscle length) with repeated measures was performed to assess neuromuscular function of the young and old adults during the isometric reference MVC and the isometric steady state following the conditioning stretch. The level of significance was set at *P* < 0.05. When significance was observed a post hoc analysis using unpaired t-tests was performed with a modified Bonferroni correction factor to determine where significant differences existed over time. Because voluntary activation values were not normally distributed, a Mann–Whitney *U*-test was employed for this particular variable. A linear regression analysis (*R*^2^) was performed to evaluate the relationship and shared variance between muscle architecture and force enhancement. A power calculation was determined to ensure there was sufficient power (1 − β = 0.82) to detect significant differences for the primary outcome measures. The tables are presented as means ± standard deviations (SD), and figures as means ± standard errors (SE).

## Results

**Figure 3:**
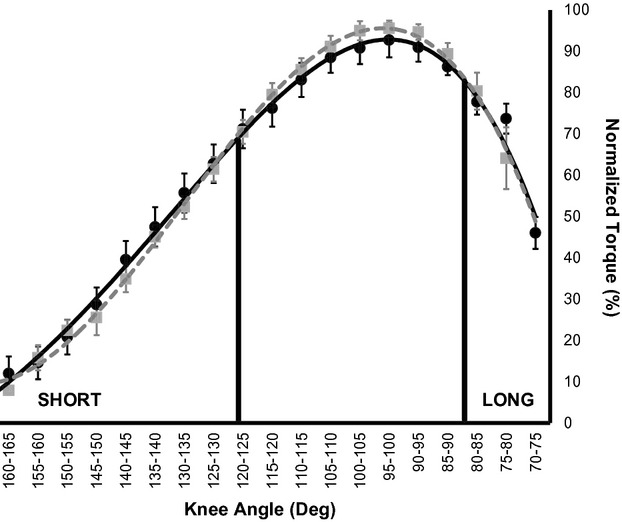
Torque-Length relationship in Young (solid black line and circles) and Old (dashed gray line and squares). Values are Means ± SE

As shown in Table 1, during isometric voluntary efforts, the old men were 37% weaker for MVC torque compared with the young men (*P* < 0.05) despite similar and equal high voluntary activation levels (≥95%, *P* > 0.05). As well, concentric strength was 32% lower in older adults (*P* < 0.05) than young. Despite, a 25% lower eccentric torque in older adults than young, the ratio of eccentric torque to isometric MVC was 17% greater in older adults (*P* < 0.05), representative of a relative age-related maintenance of eccentric strength. As shown by the T-L relationships (Fig. 3) and optimal angle of torque production during shortening (Table 1), both groups had similar T-L relationships for the knee extensors (*P* > 0.05). The older adults show the typical age-related alterations in muscle architecture characterized by 18% shorter and 22% less pennate fascicles at long muscle lengths at rest (*P* < 0.05) Table 2.

**Table 1:** Neuromuscular properties of the knee extensors

Group	Voluntary contractile properties								

	Voluntary activation (%)	Isometric strength (N·m)	Concentric strength (N·m)	Eccentric strength (N·m)	Ecc:Iso (%)	Opt. Con (deg)	Opt. Ecc (deg)	PFE short (N·m)	PFE long (N·m)
Young (*n* = 11)	95.3 ± 4.2	276.1 ± 61.5^1^	224.1 ± 51.1^1^	303.6 ± 75.7^1^	1.11 ± 0.16^1^	100 ± 6	94 ± 3^1^	0.33 ± 0.38^1^	1.05 ± 0.60^2^
Old (*n* = 11)	96.3 ± 3.0	174.8 ± 34.1	151.6 ± 39.2	225.6 ± 34.8	1.30 ± 0.13	103 ± 9	97 ± 4	0.04 ± 0.12	1.38 ± 0.56^2^

Old men had lower maximal voluntary isometric contraction (MVC) strength and concentric strength despite similar and high levels of voluntary activation. Eccentric strength was better maintained in the old relative to isometric (ratio of eccentric to isometric strength; Ecc:Iso). Optimal angle of concentric torque production (Opt. Con) was not different between groups, whereas the optimal angle of eccentric torque production (Opt. Ecc) occurred at shorter muscle lengths in old. Passive force enhancement (PFE) was higher at LONG versus SHORT muscle lengths in both groups. At SHORT the old had a lower PFE than the young, with a trend for greater PFE (*P* = 0.07) at LONG.

1 Denotes significant age difference.

2 Denotes significant length difference. (Mean ± SD).

**Table 2:** Muscle architecture measurements

	Baseline		Reference		Steady state	
			
	SHORT	LONG	SHORT	LONG	SHORT	LONG
Pennation angle						
Young	19.2 ± 3.5^1^	15.6 ± 1.8^1^	21.0 ± 3.2^1^	15.0 ± 1.6^1^	19.6 ± 2.6^1^	15.3 ± 2.5^1^
Old	13.2 ± 3.5	12.1 ± 2.6	13.8 ± 2.2	12.1 ± 1.8	13.0 ± 1.5	11.0 ± 1.1

Fascicle length						
Young	93.5 ± 12.4	107.7 ± 6.5^1^,^2^	73.7 ± 9.7	94.3 ± 12.3^2^	77.4 ± 5.8	99.0 ± 12.7^2^
Old	88.2 ± 8.41	95.6 ± 10.8	71.3 ± 6.8	81.7 ± 11.2	78.9 ± 7.5	89.3 ± 8.8

Measurements were obtained from five old and five young adults at baseline (rest), during the reference isometric contraction, and the isometric steady state following a conditioning stretch at SHORT and LONG muscle lengths. Pennation angle (deg) and Fascicle length (mm).

1 Denotes significant age difference.

2 Denotes significant difference between SHORT and LONG muscle lengths. (Mean ± SD)

### Residual force enhancement

**Figure 4:**
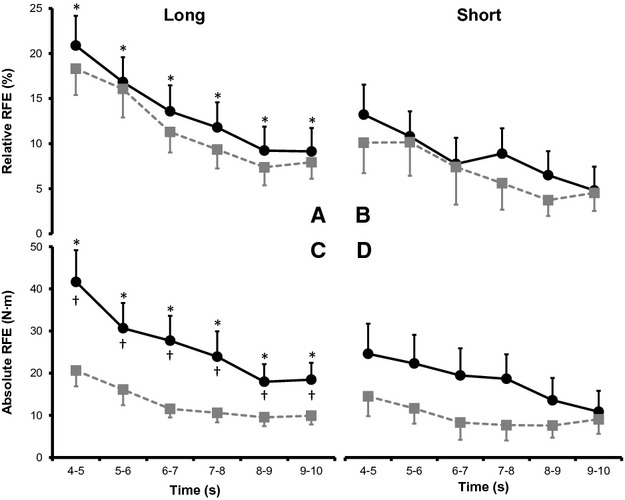
Relative RFE in Young (solid black line and circles) and Old (dashed gray line and squares) at LONG (A) and SHORT (B) muscle lengths. Absolute RFE in Young (solid black line and circles) and Old (dashed gray line and squares) at LONG (C) and SHORT (D) muscle lengths. *significance for length in both young and old for relative RFE, and only significant in young for absolute RFE, †significance for age. Values are Means ± SE.

Peak eccentric torque values during stretch were 34% and 17% greater than torque values reached during isometric contractions in old and young (*P* < 0.05), respectively (Table 1). Both old and young adults experienced the typical exponential decay of torque to isometric steady state following stretch, however, old had a longer time to reach steady-state isometric torque (3 sec) compared with young (2 sec) (*P* < 0.05). Torque values were consistently higher during the steady-state phase following stretch for both old and young, at *LONG* and *SHORT* muscle lengths compared with the reference isometric MVC (*P* < 0.05; Fig. 4).

**Figure 5:**
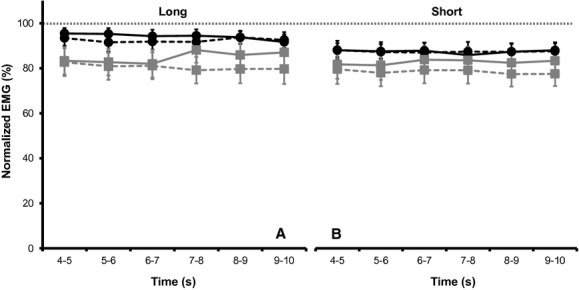
Normalized EMG for the reference (solid line) isometric and steady-state (dashed line) isometric phase at LONG (A) and SHORT (B) muscle lengths in Young (black line and circles) and Old (gray line and squares). Values are Means ± SE.

The RMS EMG of the VL was not different for the isometric reference MVC and the steady-state isometric phase following stretch at *LONG* and *SHORT* muscle lengths in both old and young, (*P* > 0.05; Fig. 5). Residual force enhancement was not different between old and young adults (*P* > 0.05) during the isometric steady-state phase following stretch and up to 6 sec succeeding stretch for both *LONG* and *SHORT* muscle lengths (Fig. 4A, B). When RFE was expressed in absolute terms (i.e., N·m) young had greater absolute RFE than old at *LONG* muscle lengths (Fig. 4C; *P* < 0.05) but groups were not different for *SHORT* (Fig. 4D; *P* > 0.05).

Passive force enhancement (PFE) was calculated as the difference between resting torque after stretch and resting torque after a reference MVC at the same final muscle length (Fig. 1), PFE was greater in old and young at *LONG* versus *SHORT* muscle lengths (Table 1; *P* < 0.05). Both old and young experienced a similar absolute level of PFE at *LONG* muscle lengths, although old showed a trend toward greater PFE than young which did not reach statistical significance (*P* = 0.07) (Table 1), whereas at *SHORT* lengths old had significantly lower PFE (*P <* 0.05). Passive force enhancement contributed 13% and 4% to total RFE at *LONG* muscle lengths in old and young, respectively, and 0.5% and 2% to total RFE at *SHORT* muscle lengths in old and young, respectively. Thus, despite similar levels of RFE and PFE in old and young at *LONG* muscle lengths, the old showed a greater relative reliance on PFE for total force enhancement.

### Ultrasound data

Pennation angles (PA) did not change (*P* > 0.05) but fascicle length (FL) decreased from rest to isometric contraction in both young and old at *LONG* and *SHORT* lengths (*P* < 0.05; Table 2). However, upon comparing the isometric reference to the isometric steady state following stretch there were no significant differences in muscle architectural changes between muscle lengths, or with respect to age (*P* > 0.05; Table 2).

### Regression analysis

There were weak, nonstatistically significant correlations and relationships between PA and FL change following stretch and RFE in old and young, respectively, *LONG* old PA = [RFE (%) = 0.33ΔPA + 14.37, *R*^2^ = 0.17, *P* = 0.49], *LONG* old FL = [RFE (%) = −0.19ΔFL + 13.41, *R*^2^ = 0.07, *P* = 0.67], *LONG* young PA = [RFE (%) = −0.66ΔPA + 7.63, *R*^2^ = 0.32, *P* = 0.19], *LONG* young FL = [RFE (%) = −0.61ΔFL + 19.96, *R*^2^ = 0.31, *P* = 0.33]. *SHORT* old PA = [RFE (%) = −0.03ΔPA + 1.68, *R*^2^ = 0.04, *P* = 0.76], *SHORT* old FL = [RFE (%) = −0.01ΔFL + 1.89, *R*^2^ = 0.00, *P* = 0.97], *SHORT* young PA = [RFE (%) = 0.14ΔPA + 8.42, *R*^2^ = 0.02, *P* = 0.82], *SHORT* young FL = [RFE (%) = −0.43ΔFL + 10.21, *R*^2^ = 0.15, *P* = 0.52].

## Discussion

In the present study, we investigated the effects of age on residual force enhancement (RFE) at long and short muscle lengths. Residual force enhancement was calculated as the increase in torque during the isometric steady state following stretch compared to a reference MVC performed prior to the stretch at the same final muscle length (Fig. 1). Baseline measures indicated that older adults were weaker for isometric and concentric strength compared with young, but that eccentric strength was better maintained (Table 1). However, because both young and old experienced a similar level of RFE at short (range: 4–13%) and long muscle lengths (range: 7–20%), the hypothesis that older adults would exhibit greater RFE compared with young, particularly at long muscle lengths owing to a greater contribution of passive force enhancement (PFE) was not supported fully (Fig. 4A, B). Interestingly, the relative contribution of PFE to total force enhancement was greater in older adults than young but only at long muscle lengths. It was hypothesized that due to age-related changes in muscle architecture, older adults would benefit from an optimized fascicle length reorganization which could contribute to enhanced force production following stretch. This hypothesis was not supported, as there were only weak, nonstatistically significant relationships between changes in muscle architecture and RFE.

### Residual force enhancement

Residual force enhancement was present in all young and old participants at long muscle lengths, however, at short lengths, a few and equal number of participants in each group experienced minimal RFE which contributed to the larger group variability at short muscle lengths (Fig. 4). Residual force enhancement in the present study was similar to values reported previously for voluntary contractions of the knee extensors (5–12%) (Hahn et al. 2010; Seiberl et al. 2010, 2011; Shim and Garner 2012) and other muscle groups (Lee and Herzog 2002; Pinniger and Cresswell 2007; Tilp et al. 2011; Power et al., 2012b,c). When a T-L relationship was constructed and normalized to peak torque amplitude (Fig. 3), both old and young had a similar relationship, with an optimal angle of torque production at ∼100° knee extension (Table 1). This was a similar finding and optimal angle of torque production to a recent investigation of older adults by Baroni et al. (2012). The T-L relationship confirms that the active stretch at short and long muscle lengths were performed on the ascending and descending limbs of the T-L relationship, respectively. However, stretch was performed over a 60° range of motion, thus moving further along the ascending limb of the T-L relationship and this could have contributed to the greater RFE at short muscle lengths compared with values at short lengths reported in the literature for the same muscle group (Shim and Garner 2012). Peak torque during the lengthening contraction was relatively greater than isometric torque and occurred at shorter muscle lengths in the old compared with the young group (Table 1). This is rather interesting given both groups had similar T-L relationships and a similar optimal angle of torque production during shortening. Whether this is due to an age-related change in stiffness of the musculotendinous unit is unknown, but warrants further investigation.

Passive force enhancement in the present study was absent or minimal at short lengths in both groups, and older adults had lower PFE compared with young. At long muscle lengths, PFE was greater than at short lengths for both groups, and there was a trend (*P* = 0.07) for older adults to have greater PFE than young. A previous investigation of the knee extensors in young adults (Shim and Garner 2012) reported greater PFE at longer muscle lengths in the knee extensors, and it is likely due to activation over the descending limb of the T-L relationship (Edman 2012). Based on the strong trend for an age-related difference in PFE at short versus long lengths, it appears older adults may require activation at longer muscle lengths for enhanced force production. In older adults, however, PFE contributed ∼10% more to RFE than young at long muscle lengths (Table 1), but was not a major contributor to RFE at short lengths. This supports our previous findings in the ankle dorsiflexors that a greater PFE in older adults is partially responsible for the age-related increase in RFE (Power et al., 2012b), and further highlights the importance of length dependence on RFE particularly in older adults. Thus, the passive components of RFE appear to be a key mechanistic contributor for the overall increase in RFE observed following stretch, particularly in older adults. Further support for a greater contribution of passive force transmitting elements in old age which could contribute to a higher RFE in older adults can be observed in the typical exponential decline in torque to isometric steady state following stretch (Edman 2012). We reported recently (Power et al., 2012b) that older adults took longer to reach an isometric steady state compared with young for the ankle dorsiflexors, and our current results for the knee extensors support these findings. Accordingly, the greater relative contribution of PFE on RFE and a longer time to isometric steady state following active stretch in older adults support strongly an increased series elastic stiffness following stretch (Edman and Tsuchiya 1996; Rassier and Herzog 2005), whether that is an active or passive mechanism or combination of both is unknown (discussed below).

### Central neural factors

Residual force enhancement in the present study can be attributed primarily to mechanical factors as neural activation (agonist; RMS EMG) levels were similar for the reference isometric MVC and steady-state MVC following stretch at short and long lengths and did not differ between old and young men. These findings are in line with other investigations of RFE for the knee extensors (Seiberl et al. 2010, 2011), although it has been suggested that neural inhibition during and following stretch contributes to an absence of RFE during unilateral knee extension (Hahn et al. 2007). An investigation of single motor unit properties following submaximal lengthening contractions showed a reduction in firing frequency while surface EMG was not different from a reference isometric contraction (Altenburg et al. 2008). However, when corrected for kinematic differences, EMG and motor unit firing rate were similar to baseline (Altenburg et al. 2009). The role of neural inhibitory influences on RFE are equivocal, such that RFE was reported to be 4–12% during a leg press task when EMG amplitude was greater following stretch during the isometric steady-state phase than the reference isometric contraction (Hahn et al. 2010). Still, it is unclear how RFE in the knee extensors can range from nonexistent (Hahn et al. 2007) upwards to 20% in the present study. A responder versus nonresponder effect has been suggested to account for varying levels of RFE (Oskouei and Herzog 2005; Seiberl et al. 2010), and perhaps, different lengthening protocols may be a factor. Stretch amplitude is an important consideration in the development of RFE (Campbell and Campbell 2011; Edman 2012; Herzog et al. 2012). In that study (Hahn et al. 2007) the knee extensors were activated on the descending limb of the T-L relationship which would explain the observed PFE, but perhaps the stretch amplitude (i.e., joint excursion) was not large enough (15–35° excursion vs. 60° in the current study) to develop discernible RFE.

### Muscle architecture

The old group showed the typical age-related alterations in muscle architecture (Narici et al. 2003; Narici and Maffulli 2010) with shorter and less pennate fascicles, and were similar to values reported previously (Baroni et al. 2012; Raj et al. 2012). As a group, fascicles shortened and pennation angles did not differ significantly from rest to activation for both short and long muscle lengths in young and old (Table 2). However, we did observe, in some subjects, fascicle length and pennation angle increased following stretch while others decreased, and this intragroup variability is consistent with a recent investigation of the knee extensors (Seiberl et al. 2010). We did not observe significant differences in pennation angle for the reference isometric condition and the isometric steady state following stretch, while fascicle length increased slightly.

We hypothesized that muscles of older adults with shorter fascicles (Narici et al. 2003; Narici and Maffulli 2010) may fall further along the descending limb of the T-L relationship than young for a given amount of stretch. If this was the case, as RFE is linearly correlated with sarcomere length on the descending limb (Edman and Tsuchiya 1996), older adults would benefit from greater stretch amplitude and because PFE is dependent highly upon stretch amplitude (Herzog and Leonard 2002), this could have contributed to the greater total RFE observed previously (Power et al., 2012b). Meanwhile at the whole muscle level, fascicle length and pennation angle did not significantly differ between the reference isometric contraction and the isometric steady state following stretch, and thus, age-related architectural changes did not contribute significantly to RFE. In accord with these findings, age-related and stretch-induced changes in muscle architecture cannot account for RFE in either age group. Using the VL as a proxy to make inferences on the architectural changes of the four heads of the quadriceps muscle group is not without error, and there could have potentially been changes during and following stretch in other component muscles which we did not observe. Other age-related changes to the musculotendinous unit such as tendon compliance (Narici et al. 2008) were not investigated in the present study and thus also cannot be ruled out.

### Potential mechanisms of RFE in an aged system

Aging is associated with a loss of isometric strength and power (Dalton et al. 2010, 2011; Power et al., 2012a), but strength is better maintained during lengthening muscle actions (Poulin et al. 1992; Roig et al. 2010; Power et al., 2012b). Impaired isometric force production in the older adult has been attributed to a reduction in the number of viable cross-bridge actomyosin interactions (D'Antona et al. 2003) and a decreased percentage of strongly bound cross-bridges (Lowe et al. 2001). In older adults, during lengthening actions, it has been suggested there is potential for greater availability of actomyosin binding sites, allowing for the recruitment of weakly bound cross-bridges into strongly bound cross-bridge states (Ochala et al. 2006, 2007). Based on these previous findings, one could argue that the muscles from older adults are operating at ‘sub-optimal’ levels during isometric actions and thus have a greater capacity for improvements in force production during and following active stretch. In reduced preparations increased muscle stiffness during stretch is a significant contributor to force enhancement, although there is little evidence for increased stiffness contributing to the residual force enhanced state (Edman and Tsuchiya 1996; Rassier and Herzog 2005). Earlier, we suggested that PFE and an increased series elastic stiffness were the major contributors to enhanced RFE experienced by older adults during and following stretch; although it is unknown if stiffness is increased following stretch in older adults. The presumed increased stiffness in older adults could be due to recruitment of the giant molecular spring titin, which is known to increase stiffness when in the presence of Ca^2+^ (Herzog et al. 2012) or even an age-related shift in titin isoforms to stiffer structures resistive to stretch.

## Conclusion

Old and young adults experienced a similar level of residual force enhancement following a conditioning stretch of the knee extensors, with higher levels at long muscle lengths. As well, there was greater passive force enhancement at long muscle lengths in both groups. Fascicle length and pennation angle changes of the vastus lateralis following stretch did not contribute to RFE in either group. Thus, age-related and stretch-induced changes in muscle architecture cannot account for RFE in either age group. Despite a lower isometric torque in older adults both groups benefited from stretch on subsequent torque production. Older adults relied more than young on PFE for total RFE, and had a longer time to isometric steady state following stretch. These findings support an increased musculotendinous stiffness following stretch in older adults, and warrants further investigation on the contribution of active and passive mechanisms contributing to RFE in older adults.
